# Comment on ‘Electric polarizabilities of CH_4_, CO, and O_2_ determined by an optical refractometer’

**DOI:** 10.1088/1681-7575/ae4c90

**Published:** 2026

**Authors:** Patrick F Egan

**Affiliations:** National Institute of Standards and Technology, Gaithersburg, MD 20899, United States of America

**Keywords:** refractive index, oxygen absorption, magnetic susceptibility, cavity deformation

The work of Zhao *et al* [1] has several problems. This Comment focuses on issues specific to oxygen. However, questions about units of magnetic susceptibility and the stability of elastic properties apply to their results for all gases.

## a. Microwave absorption:

Oxygen has weak absorption at visible and infrared frequencies, but there is a strong peak near 60 GHz [2].

Mathar’s tool hitran2refr [3] calculates refractive index as a superposition of independent oscillators. The dielectric response at visible frequencies is driven by ultraviolet oscillators (electronic transitions), which Mathar obtains from the dipole oscillator strength distribution (as pseudodipole pairs). Low frequency behavior is governed by infrared and microwave oscillators, for which Mathar uses absorption line strengths and locations from HITRAN.

Zhao *et al* [1] extrapolate one optical measurement to zero frequency using a Cauchy equation $A_{R}=A_{0}+A_{2}\sigma^{2}+A_{4}\sigma^{4}+\cdots$, with the dispersion coefficients derived from the dipole oscillator strength distributions. Egan deduced [4] a Sellmeier equation for the optical refractivity of oxygen by combining his two-color measurements with literature:

(1) $$10^{8}(n_{\text{os}}-1)=\frac{5627\;886.8}{281.0-\sigma^{2}}+\frac{197\;258.6}{45.4-\sigma^{2}},$$

at standard conditions 20 °C and 100 kPa, and σ=μm∕λ is a dimensionless frequency. A Sellmeier equation with only ultraviolet oscillators has the same low frequency behavior as a Cauchy equation.

When extrapolating an optical measurement of oxygen to zero frequency, the salient point is that (1) does not account for absorption: it has no infrared or microwave oscillator. The hitran2refr calculator provides semi-quantitative information on how much (1) might deviate from actual refractive index at low frequency. The deviation between optical refractive index $n_{\text{os}}$ and the hitran2refr calculation $n_{\text{hitran}}$ is plotted in figure 1.

The microwave measurement by May *et al* [5] was performed at $\sigma =3\times 10^{-5}$, just below oxygen’s strong microwave absorption peak. Clearly, figure 1 shows $n_{\text{hitran}}$ is higher than $n_{\text{os}}$ at this frequency. The difference in deviation at $\sigma =3\times 10^{-5}$ relative to σ≈1 is a semi-quantitative estimate of how much an optical measurement extrapolated to low frequency might deviate from May *et al* [5]. The estimate is 2.7 × 10^−3^ relative deviation. That is, because May *et al* measured just below the strong microwave absorption of oxygen, their molar polarizability is expected to be $2.7\times 10^{-3}A_{\epsilon }$ higher than what is deduced from optical measurement.

## b. Magnetic susceptibility $A_{\mu }$:

Zhao *et al* [1] use a value 0.003 449 cm^3^mol^−1^ for the magnetic susceptibility of oxygen. Their stated value appears to be in the cgs units of the CRC Handbook [6], which a recent edition gives as $\chi_{\text{cgs}}=0.003\;415\;{\text{cm}}^{3}{\text{mol}}^{-1}$. The handbook value is primarily based on the precision measurement of May *et al* [5] who reported $\chi_{\text{SI}}=0.042\;92\;{\text{cm}}^{3}{\text{mol}}^{-1}$ in SI units. The conversion between cgs and SI units is $\chi_{\text{cgs}}=\chi_{\text{SI}}∕(4\pi )$, which reproduces the handbook value. Further, May *et al* follow the convention $A_{R}=A_{\epsilon }+A_{\mu }$ with $A_{\mu }\equiv \chi_{\text{SI}}∕3=0.01431\;{\text{cm}}^{3}{\text{mol}}^{-1}$. Based on these facts, the static value $A_{\epsilon }$ of Zhao *et al* changes from the stated 3.958 19 cm^3^mol^−1^ to 3.947 33 cm^3^ mol^−1^.

## c. Two-color measurement $A_{0}$:

The two-color measurement by Egan [4] produces $A_{0}=3.957\;59(3)\;{\text{cm}}^{3}{\text{mol}}^{-1}$ at 303 K. This value is an exact numerical solution for measured data [7] and has no dependence on $A_{\mu }$. However, uncertainty in oxygen dispersion (i.e. Cauchy coefficients $\sigma^{4}$ and higher) influences the result at the few $10^{-6}A_{0}$ level. Subtracting the $A_{\mu }$ of May *et al* from Egan’s $A_{0}$ result gives $A_{\epsilon }\to 3.94329\;{\text{cm}}^{3}{\text{mol}}^{-1}$. This inferred value is $3.8\times 10^{-3}A_{\epsilon }$ lower than the microwave measurement of May *et al* Recall from above that hitran2refr predicts optical measurement should be $2.7\times 10^{-3}A_{\epsilon }$ lower than May *et al*

Egan’s two-color measurement is $1.02\times 10^{-3}A_{\epsilon }$ lower than Zhao *et al* [1]. The respective standard uncertainties are $7.2\times 10^{-6}A_{R}$ and $46\times 10^{-6}A_{R}$. So, discrepancy between the two inferred values of $A_{\epsilon }$ is about 10 times the mutual expanded uncertainty.

## d. Stability of elastic properties:

Zhao *et al* [1] model Fabry–Perot cavity deformation as having an overall compressibility 1/(3*K*) plus a mirror bending *d* induced by (slight) material mismatch between the mirror and the spacer. In past work [8], distortion in their apparatus was characterized using argon, and values *K* = 33.37 GPa and *d* = 1.6 × 10^−13^ Pa^−1^ were derived. Zhao *et al* performed a new characterization of distortion using nitrogen which yielded *K* = 30.82 GPa and *d* = −2.0 × 10^−13^ Pa^−1^. Since n−1∝p∕(3K), the larger K would yield lower refractivity. If the measurement of Zhao *et al* were reanalyzed with the previously determined *K* and *d*, their result for oxygen would decrease by $1.8\times 10^{-4}A_{\epsilon }$. The magnitude of decrease would explain one-sixth of the difference from Egan’s two-color measurement.

Further, it can be shown that when *K* of the mirror is larger than *K* of the spacer, the mirror bends out from the cavity, so *d* should be signed negative. An axisymmetric simulation is shown in figure 2 for similar conditions to Zhao *et al,* who had fused silica mirrors optically contacted to a titaniasilicate spacer. The simulation has elastic modulus *E* = 73 GPa and Poisson’s ratio *ν* = 0.16 for the mirror with *E* = 67.6 GPa and *ν* = 0.17 for the spacer; the mirror is 25.4 mm diameter by 6.35 mm thick and the spacer is 50 mm diameter by 100 mm long; the spacer bore diameter is 6 mm. The front surface of the mirror bends out from the spacer, which contributes an increase to cavity length as pressure increases and the spacer compresses. Consequently, ∣Δf∕ν∣+Δp∕(3K)>n−1 and $d\approx -5.6\times 10^{-15}\;{\text{Pa}}^{-1}$, which is more than an order of magnitude smaller than the estimate of Zhao *et al* (Here, bending refers to displacement on the front surface of the mirror. Bending is defined as the difference in displacement between the mirror center relative to the point of contact at the spacer bore. Magnitude and signage on *d* in figure 2 are consistent with Takei *et al* [9] who had mirror *K* 59% *smaller* than spacer *K*.)

It is difficult to conceive how the effective compressibility of a Fabry–Perot cavity would increase 8.3% between characterizations, or how mirror bending would change sign at the stated levels of magnitude. If monolithic glass assemblies are this unstable, worldwide advocates of the optical pressure scale have not been forthright about their system limitations.

## Figures and Tables

**Figure 1.**
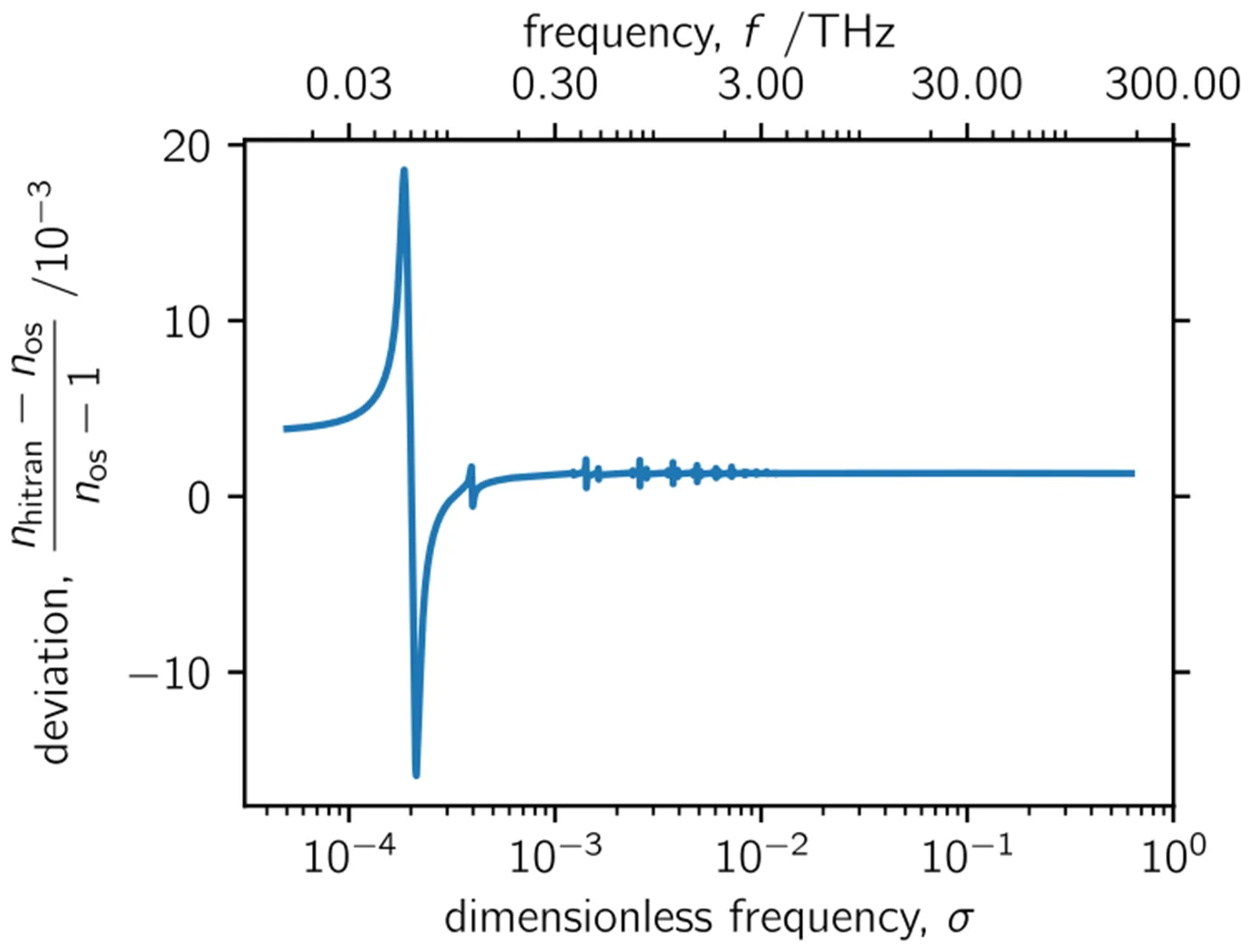
Dispersion of oxygen: deviation between an optical Sellmeier equation for refractive index $n_{\text{os}}$ by Egan [4] and a superposition of oscillators $n_{\text{hitran}}$ by Mathar [3].

**Figure 2.**
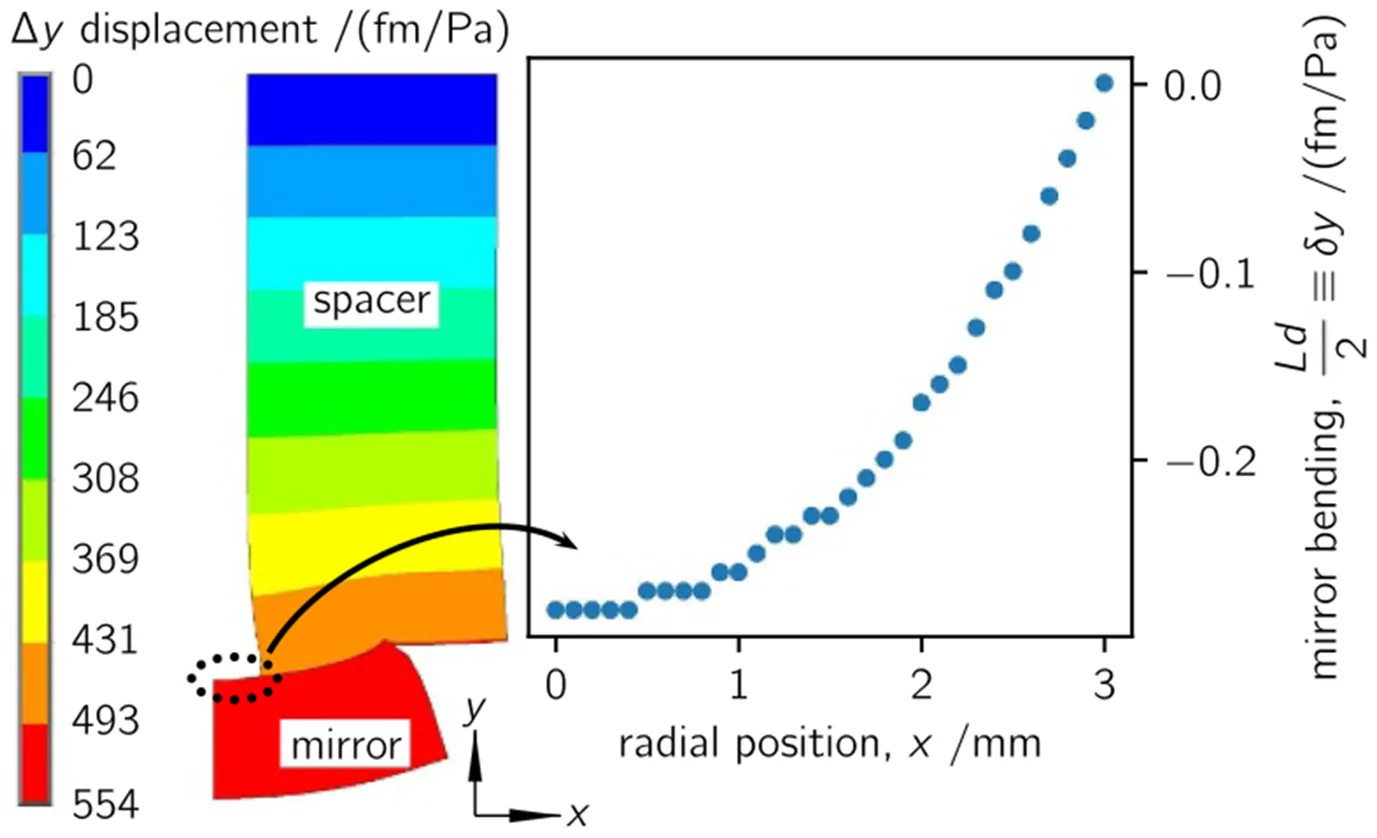
Axisymmetric model of mirror bending when mirror *K* is 4.6% larger than spacer *K*.
